# Increased expression of heat shock protein 105 in rat uterus of early pregnancy and its significance in embryo implantation

**DOI:** 10.1186/1477-7827-7-23

**Published:** 2009-03-13

**Authors:** Jin-Xiang Yuan, Li-Juan Xiao, Cui-Ling Lu, Xue-Sen Zhang, Tao Liu, Min Chen, Zhao-Yuan Hu, Fei Gao, Yi-Xun Liu

**Affiliations:** 1 State Key Laboratory of Reproductive Biology, Institute of Zoology, Chinese Academy of Sciences, Beijing 100101, PR China

## Abstract

**Background:**

Heat shock proteins (Hsps) are a set of highly conserved proteins, Hsp105, has been suggested to play a role in reproduction.

**Methods:**

Spatio-temporal expression of Hsp105 in rat uterus during peri-implantation period was examined by immunohistochemistry and Western blot, pseudopregnant uterus was used as control. Injection of antisense oligodeoxynucleotides to Hsp105 into pregnant rat uteri was carried out to look at effect of Hsp105 on embryo implantation.

**Results:**

Expression of Hsp105 was mainly in the luminal epithelium on day 1 of pregnancy, and reached a peak level on day 5, whereas in stroma cells, adjacent to the implanting embryo, the strongest expression of Hsp105 was observed on day 6. The immunostaining profile in the uterus was consistent with that obtained by Western blot in the early pregnancy. In contrast, no obvious peak level of Hsp105 was observed in the uterus of pseudopregnant rat on day 5 or day 6. Furthermore, injection of antisense oligodeoxynucleotides to Hsp105 into the rat uterine horn on day 3 of pregnancy obviously suppressed the protein expression as expected and reduced number of the implanted embryos as compared with the control.

**Conclusion:**

Temporal and spatial changes in Hsp105 expression in pregnant rat uterus may play a physiological role in regulating embryo implantation.

## Background

Heat shock proteins (Hsps) have been identified in all eukaryotic and prokaryotic organisms[1]. They may act as molecular chaperones by preventing aggregation and assisting refolding of misfolded proteins [2-4]. Hsps could be induced in response to a physiological effect or environmental effect of stress, such as elevation in temperature, oxidative stress, viral infection, nutritional deficiency, or toxic chemical exposure [5,6]. On the basis of molecular weight, mammalian Hsps have been classified into various families, including Hsp105, 90, 70, 60, and other small Hsps [4,7]. The 105 kDa protein is one of the major mammalian Hsp which belongs to the family of higher molecular mass, and is composed of 858 amino acid residues [8]. Hatayama et al. [9] demonstrated a role of this protein in protecting neuronal cells against stress-induced apoptosis in rat neuronal PC12 cells, suggesting that this protein may be a novel anti-apoptotic neuroprotective factor in the mammalian brain [10,11]. Increasing evidences indicate that Hsps could regulate cell apoptosis either by directly promoting cell apoptosis or by inhibiting apoptotic response as a chaperone of a key signaling protein [12,13]. We have demonstrated that Hsp105 was expressed in monkey testis and may play an important role in regulation of germ cell apoptosis induced by heat stress [14]. Hsp105 may function as a pro-apoptotic factor [15] or as an anti-apoptotic factor depending on cell type in mammals [16].

The evidences from our previous studies both on rhesus monkey and human being demonstrated that a relatively high frequency of apoptosis occurs in the secretory endometrium, correlated to the period of formation of implantation window [17] which was a limited period of endometrial receptivity to blastocyst stimulus[18,19]. The time surrounding the window of receptivity in the rat is referred to as the peri-implantation period and involves days 4, 5, and 6 of pregnancy. In response to implanting embryos the underlying endometrial stromal cells undergo decidualization that involves proliferation and differentiation through cell division and apoptosis [20,21]. Apoptosis is a physiological process which remodels tissue by removing expendable cells without allowing the entry of proteolytic enzymes and other harmful or corrosive substances into the surrounding tissue, and thus reducing the likelihood of an inflammatory response[22,23].

Localization of apoptotic cells in relation to the expression of apoptosis-related molecules, such as Fas/FasL, Bcl-2/Bax, and P53 have been demonstrated in the materno-fetal boundary of rhesus monkeys in pregnancy [24,25]. Apoptotic nuclei were observed mainly in the glandular cells and the blood vessel endothelial cells in decidua [26]. A transient increase in Hsp105 expression during mouse embryogenesis was observed in the embryonic tissues [9]. Human endometrium [27], deciduas [28,29] and trophoblast tissues have been also reported to be capable of expressing Hsps during the first trimester of pregnancy[30,31], however, to the best of our knowledge, no studies about an action of Hsp105 in mammalian uterus during implantation have been reported. In the present study, we have analyzed Hsp105 protein expression in rat uterus of early pregnancy, and examined the effect of injection of antisense Hsp105 oligodeoxynucleotides into the pregnant uterine horn on embryo implantation.

## Methods

### Animals

Spague Dawley rats were obtained from the Animal Facility of Institute of Zoology, Chinese Academy of Sciences. The Guidelines for the Care and Use of Animals in Research enforced by Beijing Municipal Science and Technology Commission were followed. All protocols have been approved by the Animal Care and Use Committee of Institute of Zoology, Chinese Academy of Sciences. The rats were caged in a controlled environment with a 14 hr light:10 hr dark cycle. The adult females were mated with fertile males of the same strain to induce pregnancy (day 1, D1 = day of vaginal plug positive). Pregnancy on D1–5 was confirmed by flushing embryos from the reproductive tracts. The implantation sites on D6–7 were identified by intravenous injection of 1% (w/v) trypan blue (Sigma Chemical Company, St. Louis, MO) in 0.85% (w/v) sodium chloride, according to the procedures described by Chun *et al*. and Xiao *et al*. [32,33]. In several experiments, some male rats were vasectomized, and after 14 days they were used to mate with females to induce pseudo-pregnancy (PD, PD1 = day of vaginal plug positive).

### Immunohistochemistry

In the designed time points the animals were killed by cervical dislocation under anaesthetic and the uteri were collected. In some experiments the implantation sites on day 6 and 7 were separated from the inter-implantation segments, the corrected uterine materials were fixed immediately in 10% neutral buffered formalin solution (Beijing Chemical Reagents Co. Beijing, China) overnight, and then embedded in paraffin. Serial 5 μm sections of the uterine tissues were deparaffinized and rehydrated through degraded ethanol. Antigen retrieval was performed by incubating the sections in 0.01 M citrate buffer (pH 6.0) at 98°C for 20 min, followed by cooling at room temperature for 20 min. Non-specific binding was blocked with 5% (v/v) normal goat serum (Santa Cruz Biotechnology, Inc) in PBS for 1 h. The sections were incubated with the primary antibodies against Hsp105 (sc-6241, Santa Cruz Biotechnology, Santa Cruz, CA 1: 200) in 10% goat serum overnight at 4°C. The sections were then washed three times with PBS (10 min each) and incubated with biotin labeled secondary antibody (goat anti-rabbit IgG, RT, 30 min, 1:200), After three times washes with PBS, the sections were incubated with avidin-AP complex (1:200, RT, 20 min). After three more washes, the sections were developed with Vector Red AP substrates according to the manufacturer's protocol (Vectastain ABCAP kit, Vector Laboratories, Burlingame, CA). Endogenous AP activity was inhibited by supplement of 1 mM levamisole (Sigma Corp., St. Louis, MO) into the substrate. The sections stained with Vector Red substrates were counter-stained with haematoxylin. The sections incubated with normal IgG instead of the primary antibody served as the negative controls.

### Western blot analysis

The uteri from various groups were homogenized respectively in the lysis buffer (5 mmol/L phosphate buffer, pH 7.2, containing 0.1% Triton X-100, 1 mM phenylmethylsulfonylfluoride, and 1 mg/L chymostatin), and the concentration of protein in the supernatant after centrifugation was determined by UV spectrophotometer. The sample lysates in each group were mixed with the loading buffer (62.5 mM 1,4- dithiothreitol, 5% sodium dodecyl sulfate (SDS), and 10% glycerol), boiled for 8 min, and then separated by SDS-polyacrylamide gel electrophoresis (PAGE) (50 μg total protein/lane). The separated proteins were transferred electrophoretically onto a pure nitrocellulose blotting membrane (Pall Corporation, Pensacola, FL), and then incubated with blocking buffer (3% BSA (v/v) in TBST for 1 h at room temperature. The membrane was subsequently incubated with the anti-Hsp105 antibodies overnight at 4°C, washed for three times with TBST, 15 min each time, and further incubated for 1 h at room temperature with TBST containing alkaline phosphatase-conjugated secondary antibodies, and then washed three times with TBST. After one more time of wash with TBS, then the membrane was subjected to an alkaline phosphatase color reaction by a standard method. Actin protein was used as the internal control for cytosolic protein. Band intensity was determined by Quantity One Software (Bio-Rad, Hercules, CA).

### Design of oligodeoxynucleotides specific sequence for Hsp105

The sequence(s) of oligodeoxynucleotides (ODNs) (16 nucleotides in length) were: sense oligodeoxynucleotides(S-ODNs):5'-AGCCATGTCGGTGGTT-3, antisense oligodeoxy- nucleotides (A-ODNs): 5'-AACCACCGACATGGCT-3'. The A-ODNs were complementary to bases 191–206 bp within exon I of the rat *Hsp105 *(GenBank Accession Number: NM_001011901). All the sequences were thiophosphate-modified for their long half-lives in cells. FITC/A-ODNs and FITC/S-ODNs are FITC-conjugated ODNs at the 3' end. All the ODNs were synthesized by SBS Genetech Co., Ltd. (Beijing, China). Analysis of homology between the synthesized oligomer and the rodent sequences present in the GenBank data bases (release 73.0) by the Genetics Computer Group sequence analysis software package revealed that the synthetic oligomers were fully complementary only to their own specific mRNA.

### Tracking of FITC- labeled ODNs in the tissue of rat uterus

Pregnant rats were injected with *Hsp105 *S-ODNs or A-ODNs according to the procedures described by Zhu *et al*. [34]. Tracking of FITC-labeled ODNs was performed according to the procedures described by Luu *et al*. [35]. Uterine penetration of the ODNs and cross-contamination between the two horns were assessed by injecting 10 μg (in 100 μL distilled water) of FITC-A-ODNs or FITC-S-ODNs into one horn, with either unlabeled standard control A-ODNs or S-ODNs alone into the contralateral horn of the uterus. The uteri were excised and frozen in OCT compound (QIAGEN N.V.) at 2.5 h, 24 h and 48 h after injection of the ODNs. 6 μm thick frozen sections were then analyzed under a fluorescence microscope at 488 nm.

The ODNs experiments were carried out in the afternoon on day 3 of pregnancy. The animals were divided into two groups, each group (n = 12/ODNs pair, 6 for S-ODNs and the other 6 for A-ODNs) was subject to a surgical operation and each uterine horn was injected with 10 μg of A-ODNs targeted against exon I of the Hsp105 or the corresponding S-ODNs or double distilled water (DD water). The animals were killed at 24 h and 48 h, respectively, after the operation, the uteri were fixed immediately for overnight in 10% neutral buffered formalin solution (Beijing Chemical Reagents Co. Beijing, China) and embedded in paraffin. Serial 5 μm sections of the uterine tissues were deparaffinized and rehydrated through graded ethanol for immunohistochemical analysis.

### Microscopic assessment and statistical analysis

The uterine samples from 3 rats in each group were analyzed. Experiments were repeated at least three times, from which one taken from at least three similar results was presented as a representative of the immunocytochemical data in the group. Signal intensities of Hsp105 detected by immunohistochemistry were quantified by computer-aided laser-scanning densitometry (Personal Densitometer SI; Molecular Dynamics, Inc., Sunnyvale, CA). In order to make the statistical significance of quantitative difference credible, three slides from each of six animals of each group were examined (n = 6), and 40 spots were randomly selected in every specific location of the specific cell types. The gray level of intercellular substance was considered as background. Statistical analysis was carried out with SPSS (version 10.0, SPSS Inc., Chicago, IL), and one-way ANOVA was used followed by Post-Hoc comparisons for analyzing the data in different groups. P values lower than 0.05 were considered statistically significant. To estimate specific staining in various cells of the uteri, a semi-quantitative subjective scoring was also performed by three blinded investigators using a 4-scale system with – (nil), +/- (weak), + (moderate), and ++ (strong), as described by Yue *et al*. [36]. The statistical data of Western blot from three individual experiments were analyzed by using Statistical Package for Social Science (SPSS for Windows package release 10.0, SPSS Inc., Chicago, IL). Statistical significance was determined by one-way ANOVA. Post-Hoc comparisons between groups were made using Fisher's protected least-significance-difference test. Values were means ± SEM. P values lower than 0.05 were considered statistically significant.

## Results

### Hsp105 expression in rat uterus during early pregnancy

**Figure 1:**
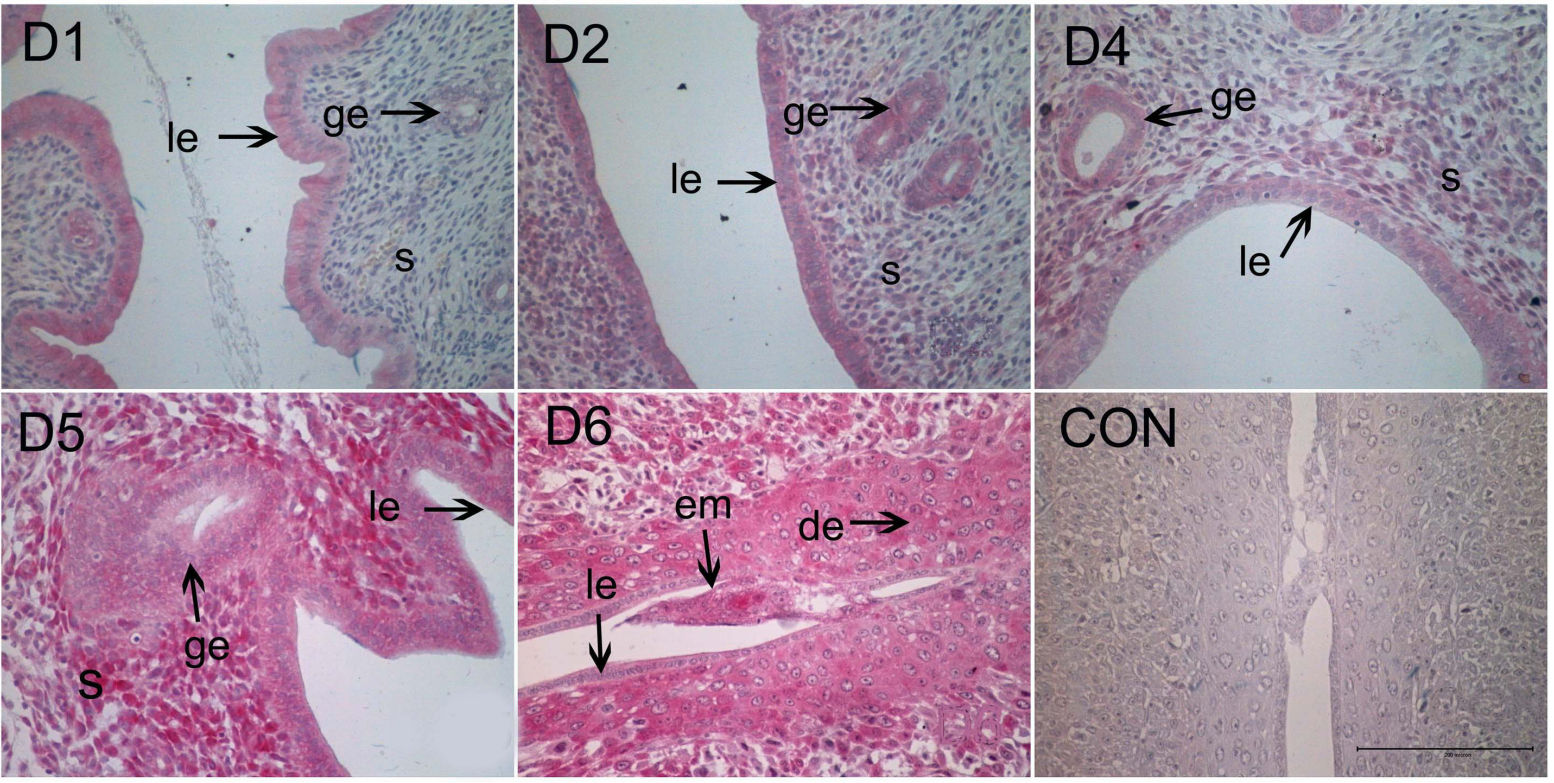
**Immunohistochemistry of Hsp105 in rat uterus during early pregnancy**. Hsp105 protein was observed mainly in the luminal epithelium on day 1 of pregnancy (D1) and moderately expressed in the luminal epithelium and the glandular epithelium from day 2 to day 3 (D2). Hsp105 staining was also detected in the stromal cells, immediately underneath the luminal epithelium on day 4 and day 5 of pregnancy (D4, D5), the staining was increased markedly on day 5 just before implantation (D5). On day 6, the protein staining was mainly observed in the implanted blastocyst and the stromal cells around the implantation site (D6), while its expression in the luminal epithelium reduced to an undetected level. CON, negtive control; le, luminal epithelium; ge, glandular epithelium; s, stroma; de, decidua; em, embryo. Bar = 200 μm.

In order to examine developmental expression of Hsp105 in rat uterus of normal pregnancy, we performed immunohistochemistry using an antibody against rat Hsp105 protein. The results showed that Hsp105 expression was mainly localized in the luminal epithelium on day 1 of pregnancy (Fig. 1, D1), and increased in the glandular epithelium on days 2 and 3 (Fig. 1, D2). On days 4 and 5, additional staining was observed in the stromal cells immediately underneath the luminal epithelium, reaching a peak level on day 5 (Fig. 1, D4, D5). The strongest expression of this protein was detected in the decidual cells adjacent to the implanting embryo on day 6 (Fig. 1, D6). Localization and average score of Hsp105 protein at the various uterine locations are summarized in Table 1.

**Table 1:** Semi-quantitative estimation of Hsp105 expression in the various uterine cells during early pregnancy

	Days of early pregnancy					
Cell Types	1	2	3	4	5	6
Luminal epithelium	+	+	+	+	++	-
Glandular epithelium	+/-	+	+	+	+++	-
Stromal cells				+	+++	-
Primary Deciduas						+++
Embryo						+++
Secondary Deciduas						+++

### Western blot analysis of Hsp105 expression in uterus during early pregnancy

**Figure 2:**
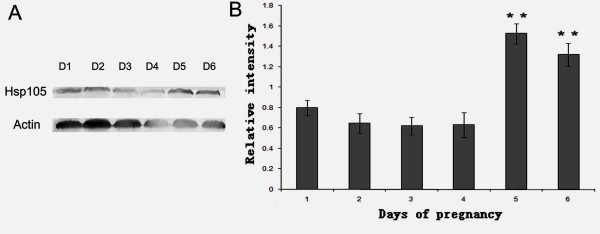
**Western blot analysis of Hsp105 protein in uterus during early pregnancy**. A: Representative Western Blot analysis of Hsp 105 protein. Actin protein was used as an internal control. B: The bar graph represents the densitometric analysis of the Hsp 105. The relative intensity was determined by the ratio of Hsp105 protein to its corresponding internal control as measured by densitometry. Data are presented as mean ± SEM (n = 3). Statistical analysis was performed using one-way ANOVA followed by the Fisher's protected least-significance-difference test. Bar with ** is significantly different from D1 of pregnancy (P < 0.01).

The quantitative change in uterine Hsp105 expression was estimated by Western blot, as shown in Fig. 2. The protein level in the uterus was increased in a time-dependent manner, the highest expression was observed on day5 and day 6, just around the time before and after implantation.

### Hsp105 expression in rat uterus during pseudo-pregnancy

**Figure 3:**
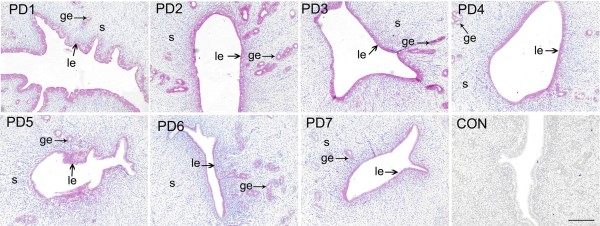
**Immunohistochemistry of Hsp105 during pseudopregnancy**. The adult male animals were vasectomized and 14 days later were used to mate with the adult females of the same strain to induce pseudo-pregnancy (pseudo-pregnancy day 1 (PD1) = day of vaginal plug positive). Hsp150 protein was mainly detected in the luminal epithelium on day 1 (PD1). The protein staining was increased in the luminal epithelium and glandular epithelium from day 2 to 3 (PD2, PD3). The protein expression was decreased from day 4 to 7 (PD4, PD5, PD6, PD7). CON, negtive control from day 2 of pseudopregancy; le, luminal epithelium; ge, glandular epithelium; s, stroma. Bar = 200 μm.

To further confirm specific expression of Hsp105 in relation to implantation, we performed an experiment with pseudopregnant rats. The protein was mainly localized in the luminal epithelium on day 1 (Fig. 3, PD1), with the staining increased in both the luminal and the glandular epithelium on day 2 and 3 (Fig. 3, PD2, PD3), sharply decreased on day 4, and remaining at a low level on day 5 to 7 (Fig. 3, PD4, PD5, PD6, PD7). No peak level expression of this protein was observed in the pseudopregnant uterus. The score of the specific cell staining for Hsp105 in the uterus during pseudopregnancy is summarized in Table 2.

**Table 2:** Semi-quantitative estimation of Hsp105 expression at the various uterine cells during pseudopregnancy

	Days of pseudopregnancy						
Cell Types	1	2	3	4	5	6	7
Luminal epithelium	+	+	+	+/-	+/-	+/-	+/-
Glandular epithelium	+/-	+	+	+/-	+/-	+/-	+/-
Stromal cells	-	-	-	-	-	-	-

### Comparison of Hsp105 protein expression in uterus between implantation site and inter-implantation segment

**Figure 4:**
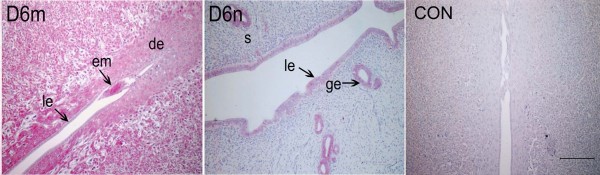
**Immunohistochemistry of Hsp105 in implantation site and inter-implantation segment**. The pregnant rats were killed on Day 6, the implantation sites (D6m) and the inter-implantation segments (D6n) were isolated for preparation of immunohistochemistry. Hsp105 expression in endometrium at the implantation site was much stronger than that at the inter-implantation segment. CON, negtive control; le, luminal epithelium; ge, glandular epithelium; s, stroma; de, decidua; em, embryo. Bar = 200 μm.

In order to know whether Hsp105 expression is related to implantation, we analyzed its expression in both implantation site and the inter-implantation segment on day 6 by immunohistochemistry. The results showed that the expression of this protein at the implantation site (Fig. 4, D6m) was much stronger than that in the interimplantation segment (Fig. 4, D6n), as summarized in Table 3.

**Table 3:** Semi-quantitative estimation of Hsp110 expression at the various uterine cells in implantation site and interimplantation segment on day 6 of pregnancy

Cell Types	Implantation site	Interimplantation segment
Luminal epithelium	-	+
Glandular epithelium	-	+
Primary decidua	+++	
Embryo	+++	
Secondary decidua	+++	

### Suppression of Hsp105 expression in pregnant rat uterus by antisense ODNs

**Figure 5:**
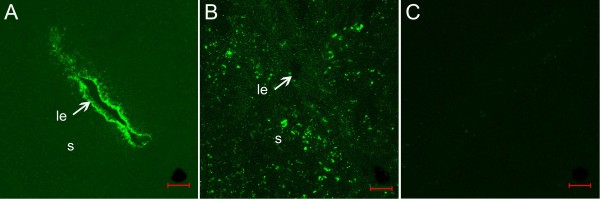
**Fluorescence micrographs of rat uteri after intrauterine administration of FITC-ODNs**. (A) The uterine horn treated with FITC- ODNs (either sense or antisense) displaying high level of fluorescence only in the luminal epithelium 2.5 hours after administration. (B) 48 hours later, a moderate fluorescence in the stromal compartment was observed after treatment with FITC- ODNs (either sense or antisense). (**C**) Uterine horn treated with unlabeled control A-ODNs displaying no fluorescence. le, luminal epithelium; s, stroma. Bar = 100 μm.

Using an A-ODNs as a blocker we examined effect of blockage of *Hsp105 *gene expression on rat implantation. To assess A-ODNs penetrating capacity, the *Hsp105 *FITC-ODNs was first injected into the uterine lumen, and then the uteri were taken for preparing sections at the indicated time points for FITC-ODNs examination by fluorescence microscopy. Strong green fluorescence representing cellular uptake of FITC-ODNs was observed in the luminal epithelium at 2.5 hours after injection (Fig. 5A). A detectable fluorescence in the underlying stroma was detected 48 hours later (Fig. 5B), indicating the penetration of *Hsp105 *ODNs into these cells *in vivo*. No fluorescence was observed in the contralateral horn treated with the unlabeled ODNs as the control (Fig. 5C).

**Figure 6:**
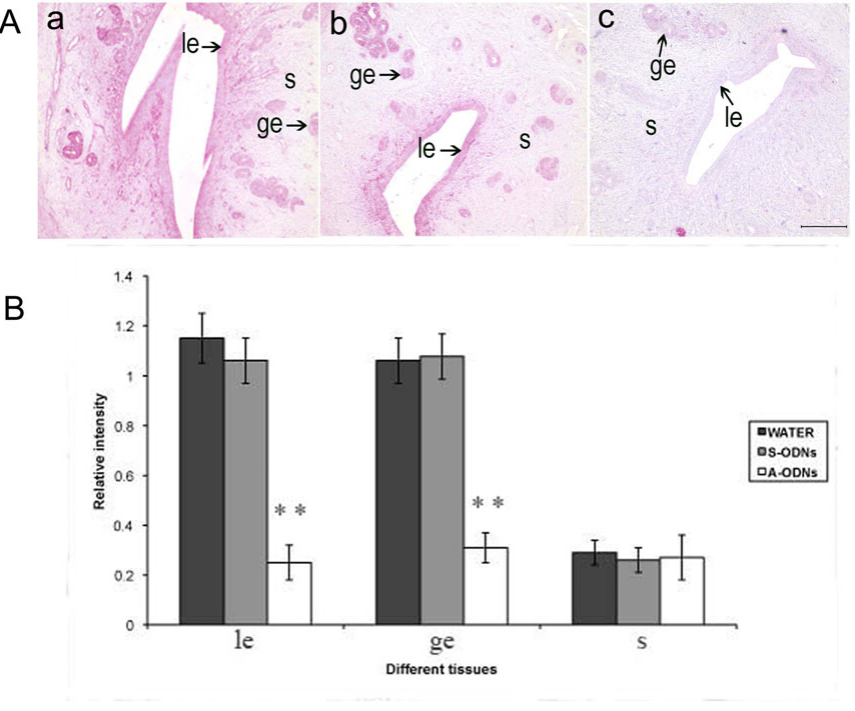
**Hsp 105 immunohistochemistry staining analysis of the pregnant rat uterus after antisense or sense ODNs treatment**. A: The figure was one representative from at least three similar independent experiment results. The immunohistochemistry staining was greatly decreased when the uteri were treated with A-ODNs (c) as compared with S-ODNs (b) and DD water (a). B: Statistical analysis of Hsp105 protein level in the uterus treated with DD water, S-ODNs or A-ODNs. Data are presented as mean ± SEM (n = 6). Statistical analysis was performed using one-way ANOVA followed by Post-Hoc comparisons. Bar with ** is significantly different from S-ODNs treated and DD water treated control (P < 0.01). le, luminal epithelium; ge, glandular epithelium; s, stroma. Bar = 200 μm.

Based on Hsp105 expression profile in the uterus, the time window of *Hsp105 *ODNs administration should be between days 3 and 5 of gestation for allowing blockage of its protein expression. The pregnant rat uteri were injected with either DD water, or *Hsp105 *S-ODNs or *Hsp105 *A-ODNs on day 3 of pregnancy, the uteri were collected 24 h and 48 h later, and then subjected to immunostaining analysis. As shown in Fig. 6A (a, b), an intensive staining was observed mainly in the luminal epithelium and glandular epithelial cells in the uterus treated with water and S-ODNs respectively. In contrast, the contralateral horn treated with A-ODNs showed only low level of Hsp105 staining on day 4, 24h after injection of ODNs (Fig. 6A (c)). A marked decrease in Hsp105 immunostaining was noted on day 5 after treatment with A-ODNs (data not shown). Statistical analysis by the computer-aided laser-scanning densitometry showed that the Hsp105 levels between the uteri treated with DD water, S-ODNs and A-ODNs were significant different in the luminal epithelium and the glandular epithelium (Fig. 6B).

### Decreasing number of implanted embryos by antisense Hsp105 ODNs treatment

**Figure 7:**
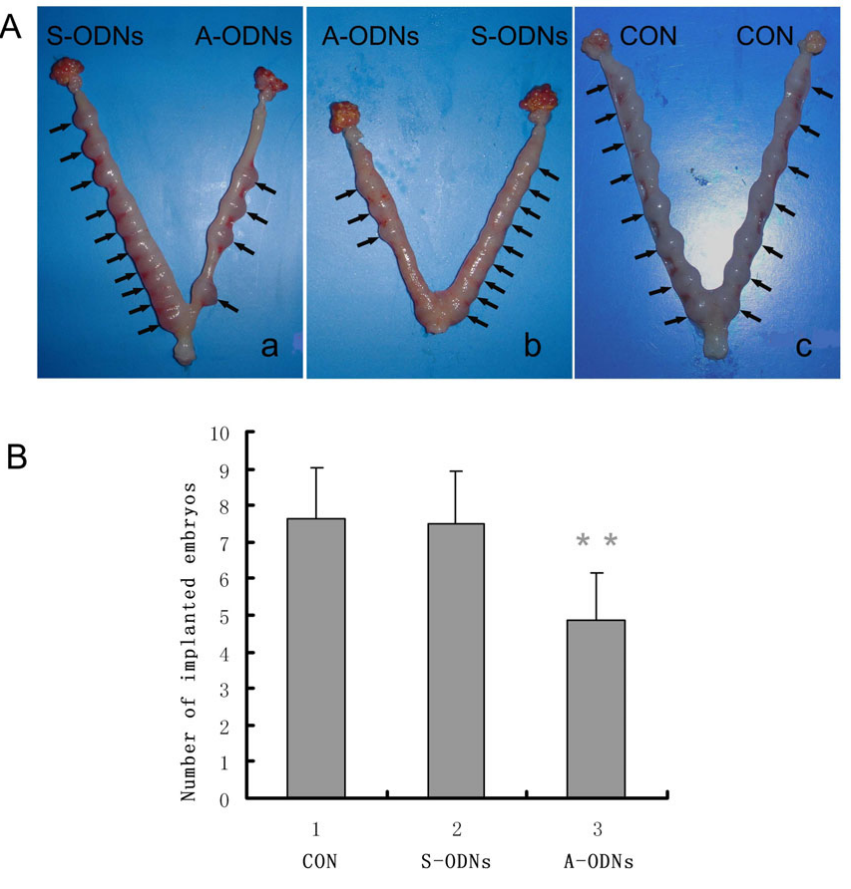
**Effect of antisense ODNs on number of implanted embryo**. **A**: Three representative uteri: (a) Pregnant rat was injected with sense *Hsp105 *ODNs in the left horn, with antisense *Hsp105 *ODNs in the right horn (10 ug in100 μl DD water) on day 3 of pregnancy; (b): Pregnant rat was injected in the left horn with antisense, the right horn with sense *Hsp105 *ODNs (10 ug in 100 μl DD water) on day 3 of pregnancy; (c): Pregnant rat was injected in both horns with DD water (100 μl). **B**: Statistical analysis of implanted embryo numbers in the uteri with the various treatments. Vertical axis represents the number of implanted embryos in the unilateral uterine horn. Data are presented as mean ± SEM (n = 8). Statistical analysis was performed using one-way ANOVA followed by Post-Hoc comparisons Bar with ** is significantly different from S-ODNs and DD water treated control (P < 0.01).

We further examined whether inhibition of Hsp105 expression could influence embryo implantation. After administration of either the antisense or the corresponding sense *Hsp105 *ODNs or distilled water into the respective unilateral uterine horns of pregnant rats on day 3, the animals were killed on day 9, and the uteri were examined for the number of implanted embryos as well as their morphological status. One representative picture of the A-ODNs- and the S-ODNs-treated uteri was shown (Fig. 7A). Ten and 9 embryos were observed in the S-ODNs-treated horns (n = 8) (a: left horn, b: right horn), while only 3 and 4 embryos (a: right horn, b: left horn) were observed in the contralateral A-ODNs-treated horns. However, all the embryos in both treated horns were normal by appearance and size. The water-injected rats contained eight to ten normal implanted embryos in each uterine horn in average (Fig. 7A(c)). No significant changes in the number of implanted embryos or the embryo normality were observed in the S-ODNs-treated horns as compared with that in the water-treated control group, indicating that the dose of ODNs used in this study was non-toxic to the embryo implantation. In contrast, as shown in Fig. 7B, a significant reduction in the number of implanted embryos in the A-ODNs-treated group was observed (60%, P < 0.01) as compared with that of the S-ODNs-treated group, but no embryo abnormality in the A-ODNs treated animals was observed.

## Discussion

The results of the present study indicate that time-dependent expression of Hsp105 in the uterine luminal, glandular epithelium and stromal cells during periimplantation period might be essential for regulation of embryo implantation. Our data also show that the presence of embryo in uterus as a stimulus may be important for increasing Hsp105 expression. If Hsp105 is involved in regulation of endometrial differentiation for embryo implantation, one may expect that a reduction of its expression could prevent acquisition of receptive state of the endometrium leading to a failure of implantation. Therefore, we designed an experiment with *Hsp105 *antisense oligodeoxynucleotides directly injecting into pregnant rat uterus at early pregnancy, which allowed us to investigate a function of this protein in the process of implantation. Technically, it would be important to do such an experiment to know the transient nature of *Hsp105 *gene expression in the uterus. In order to select an appropriate time window of ODNs administration for blockage of Hsp105 expression, we reasoned that the time window should be immediately preceding that of Hsp105 induction, i.e., between days 3 and 6 of gestation. The precise half-life of the *Hsp105 *mRNA or its protein in uterus has not yet been determined, nevertheless, the modified *Hsp105 *ODNs are known to have a half life of 24–48 hours in certain tissues [37]. Therefore, ODNs were designed for injection in the afternoon of day 3 of pregnancy, one may expect the tissue on observation to survive for the subsequent 3–4 days of gestation, for an effective suppression of the surge of Hsp105 expression. Because rat embryos were observed to be also capable of expressing Hsp105 (unpublished data), we examined a potential effect of ODNs on embryonic development by observing its normality. Therefore we selected a much later time point (Day 9) to count and examine the embryos. The statistical analysis of the difference of the numbers of implanted embryo between the antisense- and the sense ODNs-treated groups indicated that embryo implantation was indeed prohibited by the antisense ODNs (P < 0.01). These results together with the other observations suggest that treatment with antisense *Hsp105 *ODNs, but not with complementary sense ODNs, could severely impair the process of embryo implantation, but no effect on the normality of implanted embryos was observed on day 9 of pregnancy. However, Nakamura et al. just recently generated the Hsp105 knockout mice which did not appear a problem with reproduction [38], implying that Hsp105 may be not the necessary gene required for implantation in mouse. However, the authors did not specifically pay attention to examine if the animals had any implantation defect present. Hsp105 family has another two members, APG1 and APG which have shown a similar function with Hsp105, and may rescue its function in the absence of Hsp105. We have demonstrated previously that plasminogen activator is important in ovulation of rat and monkeys both in vivo and in vitro[39], however, double knockout of tPA and uPA in mice showed only 26% inhibition of ovulation could be observed[40]. Our further studies showed that mouse ovary produces not tPA and uPA, but also MMPs which have also shown to play a role in ovulation. It is possible that MMPs could rescue the function in absence of tPA or uPA. Implantation is a very complex event, which involves various processes, such as blastocyst adhesion, trophoblast invasion, decidualization and cell-to-cell interaction, controlled by a variety of molecules produced by endometrium, embryo and ovary [41]. During mammalian implantation stroma of the endometrium undergoes severe remodeling, involving apoptosis, proteolysis and angiogenesis [41,42]. Endometrial cells rapidly proliferate and differentiate to form the decidua tissue which accommodates and protects implanted embryos [43]. In our previous reports, analysis of the endometrium of both rhesus monkey and human during peri-implantation period has demonstrated that a relatively high frequency of apoptosis occurs in the secretory endometrium and is correlated to increased expression of apoptosis related molecules [25,26], while only limited numbers of the apoptotic cells were observed in the other phases of the cycle. It appears that endometrial apoptosis and the cyclic changes in endometrial growth and regression during establishment of implantation window might be regulated precisely and coordinately, not only by *Fas*, *FasL*, *BcL-2 *and *Bax *[17], but also by *Hsp105*, because the profile of these molecules is well correlated with that of the Hsp105 expression in rat uterus as demonstrated in the present study. Evidence has shown that Hsp105 is capable of enhancing cell apoptosis in mouse embryonal F9 cells[15,44] and murine embryos during embryogenesis[9,45]. On the contrary, the Hsp protein was also observed to inhibit cell apoptosis in rat testis and some experimental cell models [16,46-48]. These observations suggest that Hsp105 may be involved in regulation of murine uterine cell apoptosis. Since the cell types, species used in the individual studies were different, some unknown factors as well as cellular environment present in the various studies might determine an inhibitory or a promotional effect of the Hsp protein on cell apoptosis. However, the molecular mechanism of Hsp105 in regulating uterine cell apoptosis during rat periimplantation period remains to be further investigated. In summary, our data have demonstrated a significant increase in Hsp105 expression on day 5. It seems that the protein might be able to induce luminal cell apoptosis which in turn destabilizes epithelial barrier at implantation site and facilitates trophoblast invasion and implantation. On D6 of pregnancy Hsp105 expression was down-regulated in luminal epithelium, and upregulated in stroma cells adjacent to the embryo attachment. The dynamic changes in the level of expression of this protein during early implantation period suggest involvement of this protein in certain cellular events in luminal epithelial and stromal cells, which are essential for implantation.

## Competing interests

The authors declare that they have no competing interests.

## Authors' contributions

JXY participated together with LJX and YXL in the design of the study. The experiments were carried out by JXY, LJX, CLL, XSZ, TL, MC and FG. Data analysis was performed by JXY. The manuscript was written by JXY, FG and YXL.
